# The neuroprotective effects of Dexmedetomidine: key mechanisms focusing on neuronal programmed cell death

**DOI:** 10.3389/fneur.2026.1762900

**Published:** 2026-04-30

**Authors:** Jinxin Pan, Hui Yang

**Affiliations:** 1 Department of Anesthesiology and Operating Room, Tongjiang County People's Hospital, Bazhong, Sichuan, China; 2 Immunization Program Section, Tongjiang County Center for Disease Control and Prevention (CDC), Bazhong, Sichuan, China

**Keywords:** Dexmedetomidine, neurodegenerative diseases, neuron, neuroprotection, programmed cell death

## Abstract

In the central nervous system (CNS), programmed cell death (PCD) of neurons, is precisely regulated by various biomolecules to maintain neuronal development, establish neural structures, and maintain CNS homeostasis. Under the stimulation of pathologic factors, the abnormal cascade of PCD signals leads to irreversible damage to neuronal cells, resulting in the occurrence and progression of neurological deficits and neurodegenerative diseases (NDDs). Dexmedetomidine (DEX), a selective α2-adrenoceptor agonist, is widely used for relieving anxiety, sedation, and pain management in clinical anesthesia and critical care. A growing body of research confirms that DEX has neuroprotective effects, including reducing postoperative agitation and pain, protecting the blood–brain barrier, maintaining hemodynamic stability, minimizing neuronal damage, and alleviating neuroinflammation and oxidative stress. In this study, we will summarize the neuroprotective effects of DEX in various CNS diseases, with a focus on its regulatory role and molecular mechanisms in neuronal PCD, including apoptosis, necroptosis, pyroptosis, ferroptosis, autophagy, and parthanatos. We also explored the therapeutic potential of PCD as a target and strategy to underpin the neuroprotective effects of DEX.

## 1 Introduction

In the nervous system, neurons are the basic structure and functional units, which are responsible for receiving, processing and transmitting information, and are the structural basis of learning, memory, movement and all higher cognitive activities. Neuronal damage is a common feature of neurological disorders. In acute neurological injuries such as ischemic stroke (IS), traumatic brain injury (TBI), and spinal cord injury, rapid neuronal death caused by ischemia, hypoxia, and physical trauma is prevalent (1–3). In neurodegenerative diseases (NDDs) such as Alzheimer's disease (AD) and Parkinson's disease (PD), mechanisms such as protein homeostasis imbalance and oxidative stress contribute to progressive neuronal degeneration (4, 5). One of the common and core pathological features of these diseases is irreversible neuron damage and loss. Therefore, the search for neuroprotective strategies that can directly intervene in the neuronal death pathway and thus inhibit or delay this process is a hot research topic in the treatment of neurological diseases.

**Figure 1:**
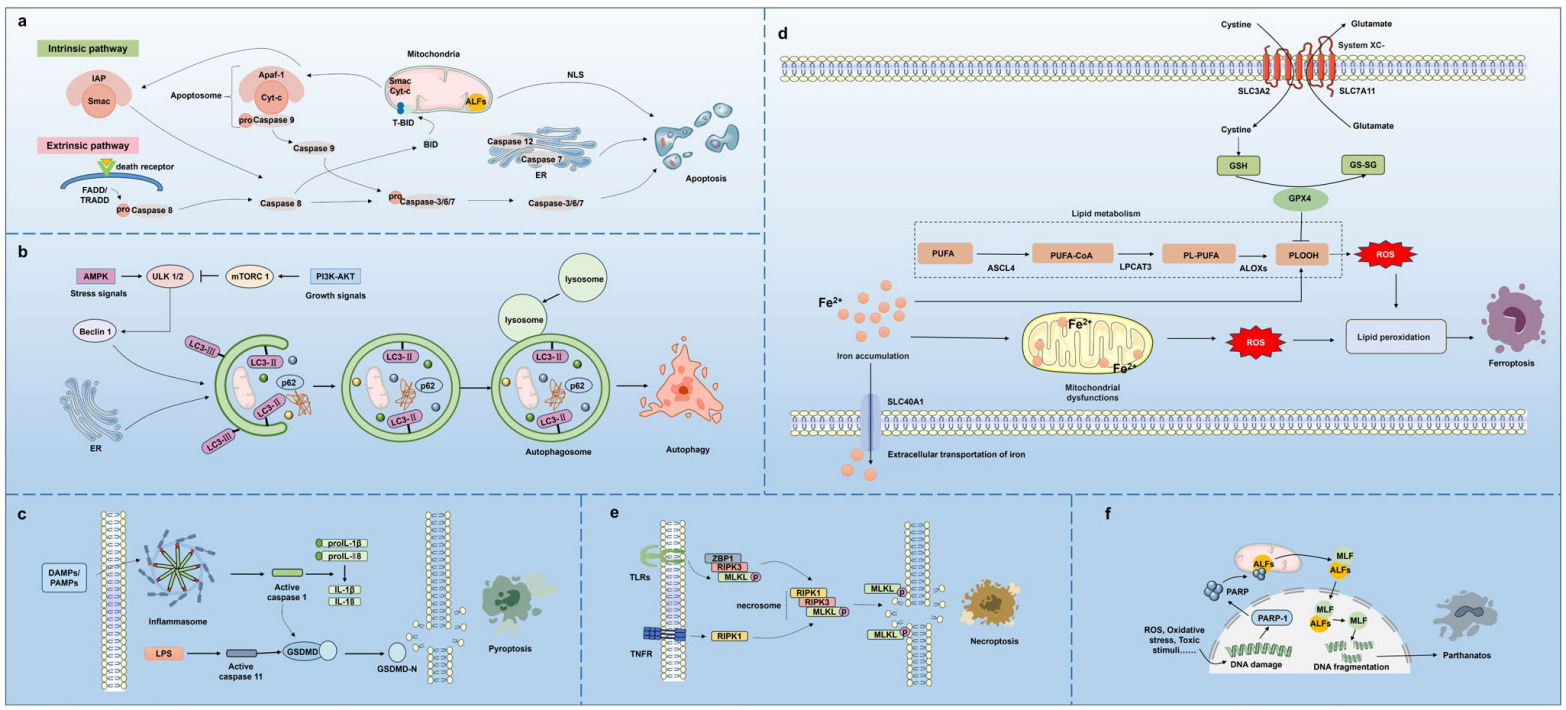
PCDs in neurons. Multiple types of PCD, including apoptosis, autophagy, pyroptosis, ferroptosis, necroptosis, and parthanatos, are involved in neuronal damage.

Programmed cell death (PCD) is a process of orderly cell death under specific conditions through gene regulation, including apoptosis, autophagy, pyroptosis, necroptosis, and ferroptosis (6). (Table 1) According to the current naming conventions of the mechanism, the term “regulated cell death (RCD)” is typically used specifically to refer to those forms of PCD that are carried out by specific molecular mechanisms and can be regulated by drugs (7). The neuronal death pathways discussed in this article fall within the framework of this RCD. PCD in neurons is ubiquitous during neurodevelopment and plays a pivotal role in maintaining functional homeostasis of the nervous system (8). Abnormal activation or suppression of PCD can lead to irreversible neuronal loss and functional impairment, thereby accelerating disease progression (9). In AD, multiple forms of PCD, including apoptosis, pyroptosis, and ferroptosis, collectively contribute to neuronal loss and pathological progression (10). In both cellular and animal models of cerebral I/R damage, pyroptosis, apoptosis, and necroptosis-related regulatory factors (e.g., NLRP3, GSDMD, caspase-8, and MLKL) are significantly upregulated, with high expression of core elements of PCD, including caspase-1, caspase-8, RIPK1, and RIPK3 (11). PCD in neurons is a complex process triggered by both endogenous and exogenous factors (Figure 1). Regulating PCD serves as a therapeutic strategy for neurological disorders. For example, rapamycin and ginkgolide B exert therapeutic effects on ischemic stroke by modulating autophagy-related signaling pathways (AMPK/mTOR, PI3K/AKT, HIF-1α, and MAPK signaling) (12). Moreover, inhibiting neuroinflammation and apoptosis can enhance neural functional recovery following SCI (13).

**Table 1:** Comparison of six modes of PCD.

Type of PCD	Morphological features	Key complex	Properties of inflammation	Characteristic mediators	Crosstalks between PCDs
Apoptosis	Cell shrinkage, nuclear condensation, apoptotic body fragmentation, nuclear membrane rupture, plasma membrane blebbing	Apoptotic bodies	No	Death receptors and their ligands, Bax, Bak, Bcl-2, AIF, caspase-3, caspase-8, caspase-9, TP53	Autophagy, pyroptosis, ferroptosis, necroptosis, parthanatos
Autophagy	Autophagic vacuolization, plasma membrane blebbing, organelle enlargement, depletion of cytoplasmic organelles	Autophagosomes and autolysosomes	No	AMPK, mTOR, ULK1, PI3KIII, BECN1, ATGs, LC3, Na+/K+-ATPase	Apoptosis, pyroptosis, ferroptosis, necroptosis
Pyroptosis	Lack of cell swelling, plasma membrane rupture, bubbling, moderate chromatin condensation, and fragmentation	Pyroptotic bodies	Yes	NLRP3, ASC, caspase-1/11, GSDMD, IL-1β, IL-18	Apoptosis, autophagy, ferroptosis, necroptosis
Ferroptosis	Small mitochondria, reduced number of mitochondrial cristae, elevated mitochondrial membrane density, increased rate of mitochondrial membrane rupture	Mitochondrial complex I and III	No	System XC-, GPX4, SLC7A11, ACSL4, lipid ROS	Apoptosis, autophagy, pyroptosis, necroptosis
Necroptosis	Cell swelling, plasma membrane rupture, moderate chromatin condensation	Necrosome	Yes	Death receptors, TLRs, TCR, RIPKs, MLMK	Apoptosis, autophagy, pyroptosis, ferriptosis
Parthanatos	Chromatic condensation, large DNA fragment formation, lack of apoptotic bodies and small-scale DNA fragments, loss of membrane integrity, lack of cell swelling	AIF/MIF complex	Yes	PARP1, AIF, MIF	?

DEX, a widely used α2-adrenergic receptor agonist, exerts sedative and analgesic effects by specifically binding to α2 receptors in the brain and peripheral tissues (14). DEX is clinically used for sedation, analgesia, and delirium reduction in intensive care unit (ICU) patients undergoing mechanical ventilation and surgical procedures (15, 16). DEX has neuroprotective effects through multiple mechanisms, including suppressing inflammatory responses, reducing cell death and autophagy, protecting the blood–brain barrier (BBB), and stabilizing cellular structures (17, 18). In various neurological injury models, DEX effectively regulates the expression of apoptosis-related proteins, thereby significantly decreasing the number of apoptotic neurons (19–21). Upon binding to α2 receptors, DEX negatively regulates the p38/MAPK and activates Nrf2 signaling pathways, thereby increasing intracellular antioxidant enzyme levels and alleviating cerebral I/R injury (22). Furthermore, DEX has been shown to improve myocardial I/R (23) and sepsis-associated organ damage (24).

In this review, we will summarize the neuroprotective effects of DEX in different central nervous system (CNS) disorders by focusing on PCD.

## 2 Neuroprotective effects of DEX

DEX has exhibited neuroprotective effects across diverse CNS disorders, including IS, TBI, and intracerebral hemorrhage (ICH) (Table 2).

**Table 2:** Clinical studies on the therapeutic effects of Dexmedetomidine in CNS diseases.

CNS diseases	Clinical parameters	Patients' source	Study type	control	DEX group	outcomes	Strength of evidence	Limitations	Ref.
IS	*n* = 2828, age >18, ICU patients	MIMIC-IV database (single-center, Boston, USA)	Retrospective cohort study	without DEX	*n =* 646	Significantly reduce mortality rates in ICU(90 days)	Large sample size; reproducible data source		(29)
	*n =* 2816, age >18	MIMIC-IV database (single-center, Boston, USA)	Retrospective cohort study	*n =* 2335	*n =* 407	Improve the prognosis of ICU patients and improve the 180-day survival rate	Large sample; long-term (180-day) outcome; Use of PSM and Cox regression; subgroup analyses performed (e.g., gender, age, Charlson score)	Single-center database; potential unmeasured confounding; treatment not standardized	(30)
	*n =* 141, age >18	China	RCT	*n =* 71, saline	*n =* 70, 0.5 μg/kg 10 min before surgery, infusion at 0.1 μg/kg/h until 72 h postoperatively	Significantly reduced 7-day CHS occurrence after mechanical thrombectomy in AIS patients and lowered postoperative pain scores.	Low bias; Safety profile assessed	Single-center; relatively small sample size; Short-term primary outcome	(32)
	*n =* 47, aged 68–84	Japan	RCT	Saline	*n =* 24, 1.0 μg/kg/h for 1h then adjust to 0.2 μg/kg/h	Low-dose dexmedetomidine improves hemodynamic stability during emergence and recovery from general anesthesia in patients receiving carotid endarterectomy.	Low bias	Single-center; small sample size; outcome not directly neurofunctional	(33)
TBI	*n =* 60, aged 18–60	India	RCT	*n =*30, lidocaine, 2 mg/kg	*n =* 30, 0.5 μg/kg	Significant decrease in MAP and CPP	Prospective, blinded design; Monitored ICP/CPP	Single-center; small sample; excluded patients with elevated ICP; No long-term outcome	(39)
TBI	*n =* 196,aged 40–65, undergoing PDT	China	RCT	*n* = 66, sufentanil infusion 0.3 μg/kg for 10 minutes, then adjusted to 0.2–0.4 μg/kg/h	0.5 μg/kg(*n* = 62) or 1 μg·kg(*n* = 68) for 10 minutes, then adjusted to 0.2–0.7 μg/kg/h	Provide the desired attenuation of the hemodynamic response without increased adverse events	Prospective design; exploration of two doses	Single-center; relatively small sample size	(40)
	*n =* 60, aged 18–75,	China	RCT	*n =* 30, propofol, 0.5–2.0 μg/kg/h	*n =* 30, 0.2–0.5 μg/kg/h	Reducing the duration of ICU stay and ventilator dependency, enhancing cerebral oxygen metabolism, and attenuating the infiltration of inflammatory factors	Low bias	Single-center; small sample size	(41)
	*n =* 90, age>18	China	Retrospective cohort study	*n =* 45, propofol 2 mg/kg/h or midazolam 0.1 mg/kg/h	0.8μg/kg then 0.25–0.75μg/kg/h	A preventive effect on PSH	Explored an important clinical complication	Single-center; small sample; retrospective; no long-term neurofunctional outcome	(43)
	*n =* 90, average age of 34.22 ± 9.18	China	RCT	*n =* 30, pethidine, morphine, diazepam, etc. ; *n =* 30, propofol, 0.5-2.0mg/kg,then 1–3mg/kg/h for 72h	*n =* 30, 0.5–1.0μg/kg, then 0.2–0. 6μg/kg/h for 24h	The sedation efficacy of DEX was superior to propofol, and was able to control excessive stress response better, and with more effect on blood pressure	Low bias	Single-center; small sample size	(44)
	*n =* 60, aged 18-65	Iran	RCT	*n =* 30, haloperidol, 2.5 mg for 10 mins every eight hours	*n =* 30, 0.5 μg/kg every other day	The patients in the dexmedetomidine group were calmer and experienced less delirium	Direct comparison vs. haloperidol	Single-center; small sample; non-robust randomization; No long-term follow-up	(45)
	*n =*208, aged 20–80	China	RCT	Routine symptomatic treatment or but (4 mg in 48 ml saline)	DEX:1 μg/kg, followed by 0.2–0.7 μg/kg/h. ; DEX+But: the same dose used alone	More stable vital signs, improved patient consciousness levels, reduced stress responses, enhanced pain and agitation scores, and better prognosis	Relatively large sample size; explored combination therapy	Single-center; long-term functional outcomes not reported	(46)
ICH	*n =* 338, aged >18	China	RCT	*n =* 171, standard treatment	*n =* 167,remifentanil 0.025 or 0.05 μg/kg/min and Dex 0.2–0.6 μg/kg/h as a supplement	Significantly increased the SBP control rate at 1 h posttreatment	Multicenter; large sample size	Inclusion of SAH may confound results; single-blinded; complex regimen	(51)
	*n =* 60, aged 21–72	India	RCT	*n =* 30, 1.5 mg/kg bolus followed by 1.5 mg/kg/h infusion	*n =* 30, 1 μg/kg for 10 min then 0.5 μg/kg/h	Reduce the incidence of adverse respiratory events and decrease the use of supplemental sedatives	Prospective, randomized; safety-focused	Single-center; Only good-grade SAH included; anesthetist not blinded; No long-term outcome	(52)

ICU, intensive care unit; DEX, dexmedetomidine; CHS, cerebral hyperperfusion syndrome; AIS, acute ischemic stroke; PDT, percutaneous dilatational tracheostomy; MAP, mean arterial pressure; CPP, cerebral perfusion pressure; PSH, paroxysmal symplastic hyperactivity; SBP, systolic blood pressure; RCT, randomized controlled trial.

### 2.1 IS

As the most common type of stroke, IS constitutes approximately 80% of all cases (25). Currently, the most effective treatment strategy involves restoring blood flow to ischemic brain tissue through medication or surgery. However, the reperfusion process may cause more severe damage to brain tissue. Ischemia–reperfusion injury is a critical component of the pathophysiology of IS (26). Numerous studies conducted over the past decade have established the neuroprotective potential of DEX in animal models of IS (27, 28). Consequently, clinical research on the neuroprotective effects of DEX in IS has become a focal point in recent years. A large retrospective study (with 2,828 cases) based on the MIMIC-IV database showed that critically ill patients with IS treated with DEX in the ICU had lower 90-day mortality and did not have an increased risk of bradycardia (29). Another retrospective study (enrolling 2,816 cases) similarly revealed that DEX sedation was associated with reduced 28-day mortality among critically ill patients admitted to the ICU and effectively lowered 180-day mortality rates in discharged patients (30). This study performed multiple factor subgroup analyses, including sex, age, Glasgow Coma Scale (GCS) score, and comorbidity (Charlson score). The results showed that DEX was more effective in improving 180-day mortality in men and in patients aged ≥65 years. DEX did not improve 180-day mortality in patients with Charlson score ≤ 6, but provided a stronger clinical benefit in patients with multiple comorbidities (30).

For acute IS (AIS) patients, endovascular thrombectomy is a standard treatment, but it may lead to severe adverse outcomes, such as cerebral hyperperfusion syndrome (CHS) (31). A single-center RCT study from China (*n* = 141) reported that the use of DEX during the perioperative period for endovascular thrombectomy in patients with AIS significantly reduced the incidence of CHS and markedly alleviated postoperative pain on days 1 and 3 (32). However, the study also reported that DEX intervention had no significant effect on the National Institutes of Health Stroke Scale (NIHSS) score at 24 h after surgery, the total length of hospital stay, the length of ICU stay, and 30-day all-cause mortality (32). The investigators noted that this may be related to the fact that the sample size calculation in this study was based only on detecting differences in the incidence of CHS, and its sample size was not sufficient to detect differences in neurological function. Another study from Japan demonstrated that intraoperative DEX administration effectively alleviated stress responses, exerted sedative effects, and improved pain management in IS patients undergoing carotid endarterectomy (33). These findings indicate that DEX serves as an effective sedative for ICU-admitted IS patients, increasing survival rates in both the short term and long term. For surgically treated IS patients, perioperative DEX infusion significantly reduces postoperative complications and provides pain relief. However, the precise long-term neuroprotective value of DEX in IS patients still needs to be verified by large-scale trials with functional outcome as the primary endpoint and adequate sample size.

### 2.2 TBI

TBI is a disease with high morbidity and mortality rates and is caused primarily by acute or blunt trauma. Most patients present with closed head injuries (34). The excessive inflammatory response and apoptosis following TBI are the main causes of secondary neuronal damage, and there remains a dearth of effective preventive and therapeutic approaches in clinical settings (35, 36). In recent studies, it has been revealed that DEX exhibits neuroprotective properties in both *in vivo* and *in vitro* TBI models, with mechanisms involving the regulation of inflammation and neuronal apoptosis (35, 37, 38). As DEX is commonly used as a sedative in ICU and surgical patients, increasing research has focused on its neuroprotective effects in TBI patients.

Studies have shown that, compared with lidocaine, DEX injection significantly reduces the mean arterial pressure (MAP) and cerebral perfusion pressure (CPP) in critically injured TBI patients undergoing mechanical ventilation or intracranial pressure (ICP) monitoring, thereby improving patient prognosis (39). In TBI patients undergoing percutaneous tracheotomy (PDT), DEX (1 μg/kg for 10 min, then adjusted to 0.2–0.7 μg/kg/h) significantly reduced both the duration and total dose of anesthetic use compared with intraoperative sufentanil administration, while also decreasing the cough reflex, tachycardia, and hypertension rates (40).

In terms of overall sedation management in the ICU, several studies have compared the effects of DEX and other sedatives. Compared with propofol sedation, DEX sedation significantly reduced the duration of ICU stay, accelerated consciousness recovery, enhanced cerebral oxygen metabolism, and lowered inflammatory cytokine (e.g., IL-6) levels in severe TBI patients (41). Excessive sympathetic nervous system activation following acute TBI may cause secondary brain damage, significantly affecting prognosis (42). DEX sedation reduces postoperative paroxysmal sympathetic hyperactivity (PSH) caused by neurological injury in severe TBI patients compared with other sedatives (propofol/midazolam) (43). Another study similarly reported that DEX demonstrated superior sedative effects to those of propofol in moderate-to-severe TBI. The DEX group presented elevated plasma β-endorphin levels during the early stages of brain injury, indicating its positive role in early stress regulation (44). Furthermore, a comparative study of haloperidol and DEX during ICU treatment in TBI patients revealed that DEX injections (0.5 μg/kg) administered every other day resulted in greater sedation and significantly reduced the incidence of delirium (45). Wang et al. compared the sedative effects of single DEX and butorphanol, and their combination in TBI patients. The results showed that the combination of DEX and butorphanol maintained more stable vital signs, improved consciousness levels, reduced stress responses, and increased pain and agitation scores, with a better overall prognosis (46). Clinical studies have shown that DEX has neuroprotective effects on TBI patients.

Compared with other sedatives, such as propofol and remifentanil, DEX delivers safer, and more effective sedation for TBI patients requiring ICU admission or surgical intervention. It has shown potential advantages in reducing inflammation, inhibiting PSH, reducing secondary brain injury, and reducing delirium. The current evidence is mostly single-center and small-sample trials, which focus on long-term neurological prognosis indicators, limiting the comprehensive judgment of the neuroprotective value of DEX in TBI. Future large-scale multicenter RCTs with long-term functional recovery as the primary endpoint in a broader TBI population are needed to clarify their clinical positioning.

### 2.3 ICH

ICH accounts for 10%~15% of all stroke cases, with high morbidity and mortality (47). The massive amount of cell death caused by ICH is a key factor in neurological deficits, and no effective treatment has been found to reduce post-ICH cell death (48). Research has demonstrated that DEX exerts therapeutic effects on cerebral hemorrhage by inhibiting NLRP3 activation, modulating microglial polarization, and promoting BBB repair (49, 50). Clinical studies have further validated the therapeutic efficacy of DEX in ICH. A trial of an intensive blood-pressure-lowering strategy in patients with spontaneous ICH conducted at 14 centers in China found that the addition of DEX to remifentanil to standard care significantly improved the rate of control of systolic blood pressure and reduced agitation within the 1 h after admission (51). This study is a composite intervention program, and it is difficult to isolate the effect of DEX alone. The primary endpoint was short-term blood pressure control, and the impact on key outcomes such as hematoma expansion and long-term neurological recovery is unclear.

Subarachnoid hemorrhage (SAH) is a severe form of stroke that may also trigger or co-occur with other types of cerebral hemorrhage, such as intracerebral hemorrhage or intraventricular hemorrhage. Compared with the use of propofol, the use of DEX during cerebral angiography in SAH patients significantly reduces the incidence of adverse respiratory events and decreases the need for additional sedatives (52). Therefore, DEX is the preferred sedative in the clinical management of ICH patients. Preliminary evidence shows that DEX has a certain role in blood pressure management and sedation in the acute phase of ICH and SAH patients, especially for rapid blood pressure control and respiratory safety. However, whether it can really improve the neurological outcome of patients with ICH/SAH remains to be verified by large multicenter RCTs in the future.

Based on the above clinical evidence, it IS found that DEX has many application potentials in the acute management of acute brain injuries (including IS, TBI, and ICH). In addition to better sedation, DEX may help stabilize hemodynamics, reduce the risk of specific perioperative or ICU complications (e.g., CHS, PSH, delirium, respiratory depression), and be associated with improved survival in some patient populations. Therefore, although the neuroprotective mechanism of DEX is well supported in preclinical studies (17, 18), its exact clinical neuroprotective value, defined by improved long-term neurological function, is still not fully confirmed by high-quality clinical studies.

## 3 Effects and mechanism of DEX on the PCD of neurons

At the molecular level, the protective effects of DEX on CNS diseases may stem from its ability to modulate multiple PCD pathways, including apoptosis, autophagy, pyroptosis, ferroptosis, necroptosis, and parthanatos (Table 3).

**Table 3:** Preclinical mechanistic studies on the neuroprotective effects of dexmedetomidine in CNS injury and disease models.

Diseases	Model types	Main outcome (molecular pathway)	PCD processes	Strengths of evidence	Limitations of evidence	Ref.
IS	OGD/R, neuro-2a cells	Attenuates Bax translocation from cytosol to mitochondria, stabilizes mitochondrial membrane potential (MMP), reduces cytochrome c release, and inhibits caspase −9, −3, −6 activation	Apoptosis	Elucidates the complete mitochondrial apoptotic pathway	Lack of *in vivo* validation	(55)
IS	OGD/R (SH-SY5Y)	Binds to and activates α2-AR, upregulates the Nrf2/ARE pathway (Nrf2, HO-1, NQO1, SOD), improves cell viability, reduces oxidative stress and apoptosis. Silencing Nrf2 reversed the protective effects of DEX	Apoptosis	Establishes causal relationship in the α2-AR/Nrf2 pathway	Lack of *in vivo* validation	(61)
Cerebral I/R	MCAO rats; OGD/R-induced primary neurons	Improves neurological deficit, reduces brain edema and infarct volume. Downregulates ER stress markers (GRP78, p-PERK, CHOP) and inhibits the downstream apoptotic protein Caspase-11 and Cleaved-caspase-3	Apoptosis	Reveals a novel ERS-dependent apoptotic pathway (PERK-CHOP-Caspase-11)	Lack of direct causal verification of the PERK-CHOP axis (e.g., inhibitors).	(66)
Cerebral I/R	MCAO/R, rats	Upregulates Sigma-1 receptor (Sig-1R) expression on the ER, increases GRP78, and inhibits pro-apoptotic ER stress proteins (CHOP, p-JNK, Caspase-3). Sig-1R inhibitor partially reversed these effects	Apoptosis	Reveals a novel Sig-1R-mediated anti-ERS apoptosis mechanism	Single observation time point	(67)
Cerebral I/R	MCAO, rats	Improves neurological function and reduces neuronal apoptosis. DEX upregulates miR-381, which targets and inhibits IRF4, leading to reduced IL-9 expression and attenuated inflammatory response	Apoptosis	Connects miRNA regulation, inflammation, and apoptosis	Unassessed neurological function	(72)
Cerebral I/R	MCAO, mice	Activates autophagy via HIF-1α/Beclin1 pathway, promoting microglial M2 polarization and protecting against neuronal apoptosis.	Autophagy, apoptosis	Elucidates the link between autophagy regulation and immune microenvironment remodeling	Causal sequence between pathways is not fully disentangled	(82)
Cerebral I/R	MCAO, mice	Upregulates HIF-1α, Bcl-2; downregulates Beclin1	Autophagy, apoptosis	Simultaneously examines markers of both autophagy and apoptosis	Insufficient mechanistic explanation of how HIF-1α coordinates both processes	(83)
Cerebral I/R	MCAO, rats	Downregulates JNK1, Beclin1, LC3-II, caspase-3	Autophagy	Suggests JNK as a potential common upstream target	Does not verify the necessity of JNK	(84)
Cerebral I/R	MCAO, rats	Inhibits miR-199a expression and downregulates Beclin1, LC3-II, caspase-3, p62	Autophagy	Reveals a nodal role for miRNA in regulating autophagy	Lack of direct functional manipulation of miR-199a	(85)
Cerebral I/R	OGD/R HT22 cell	Increases SIRT3 expression, enhances autophagy activity, and decreases the levels of LDH and H_2_O_2_, increases the levels of ATP and MMP	Autophagy	Identifies SIRT3 as a potential key mediator	Lack of *in vivo* validation	(88)
Cerebral I/R	MCAO, mice	Decreases MDA, Fe^2+^ and TFR1; increases GSH, SLC7A11, GPX4. Relieves mitochondrial damage	Ferroptosis	Uses Nrf2 inhibitors	Not combined with other PCDs	(102)
Cerebral I/R	MCAO, mice	Reverses MCAO-induced downregulation of four HIF-1 pathway-related ferroptosis genes (HMOX1, STAT3, CYBB, TLR4) and GPX4. Reduces cell death, neurobehavioral deficits, inflammation (TNF-α, IL-6), and oxidative stress (MDA, GSSG)	Ferroptosis	Combines bioinformatics prediction with *in vivo* validation, suggesting a novel potential pathway	Primarily correlative	(103)
Cerebral I/R	MCAO mice; OGD/R-induced SH-SY5Y cells	Inhibits the SOX9/DMT1 axis. SOX9 upregulation or DMT1 overexpression abolishes DEX's protective effects	Ferroptosis	Elucidates a complete pathway from transcription factor SOX9 to iron transporter DMT1	Less exploration of the specific mechanism by which DMT1 leads to ferroptosis downstream	(107)
TBI	weight-drop model, rat	Improves neurological function, reduces brain edema and neuronal apoptosis. Upregulates Nrf2 and downstream factors (HO-1, NQO-1), downregulates inflammatory cytokines (TNF-α, IL-1β, IL-6, NF-κB) and pro-apoptotic proteins (cleaved caspase-3, Bax)	Apoptosis	Integrates anti-inflammatory, antioxidant, and anti-apoptotic effects	Lack of causal verification using Nrf2 knockout/inhibitors.	(36)
TBI	controlled cortical impact (CCI), mice	Improves neurological and motor function, reduces brain edema and blood-brain barrier disruption. Attenuates ER stress marker expression and ER stress-related neuronal apoptosis	Apoptosis	Multi-level assessment with objective behavioral tests	Primarily correlative	(37)
TBI	Feeney weight-drop model, mice	Inhibits ROS, activates the Nrf2/HO-1 pathway, and reduces the expression of LC3 and Beclin-1	Autophagy	Links antioxidant effects to inhibition of excessive autophagy	Primarily correlative	(19)
Glutamate-induced cytotoxicity	Glutamate-induced PC12 cell injury	Alleviates loss of mitochondrial membrane potential, reduces intracellular Ca^2^? overload, enhances SOD activity, decreases levels of ROS and MDA. Downregulates expression of Bax, cytochrome c, caspase-3/9, and upregulates Bcl-2	Apoptosis	Systematically evaluates multiple key nodes of oxidative stress and mitochondrial apoptosis	Does not explore crosstalk with other PCDs; lacks *in vivo* validation	(56)
ICH	autologous blood injection, rats	Reduces brain water content, neurological deficits, and neuronal apoptosis. Increases antioxidant enzyme activities (GSH, SOD), decreases MDA. Activates Nrf2/HO-1/NQO1 pathway	Apoptosis	Combines *in vivo* and *in vitro* evidence, confirming the central role of Nrf2	Does not explore other key post-ICH processes	(62)
Cardiac surgery	Cardiopulmonary bypass model, rats	Reduces plasma levels of neuronal injury markers (S100β, NSE) and neuronal apoptosis in hippocampus and cortex. Lowers inflammatory cytokine (IL-6) levels. Inhibits phosphorylation of JAK2 and STAT3	Apoptosis	Focuses on a clinically relevant model with translational significance	Unassessed neurological function	(68)
Systemic inflammation	LPS, rat	Reduces pro-inflammatory cytokines and apoptosis-related proteins. Inhibits the p38 MAPK/c-Myc/CLIC4 pathway	Apoptosis	Validates the necessity of the p38 MAPK pathway at the cellular level	LPS is a systemic model, limited in mimicking specific brain neuroinflammation	(70)
Anesthetic neurotoxicity	Propofol treatment, mouse/HT22 cells	Increases DNMT3A/3B expression, enhances methylation of the miR-377-5p promoter, thereby downregulating miR-377-5p and upregulating its target Arc	Apoptosis	Reveals a complete novel pathway from epigenetics to miRNA to target gene	Generalizability to classic CNS diseases needs validation	(71)
Anesthetic neurotoxicity	Ropivacaine, PC12 cells	Reverses ropivacaine-induced reduction in PC12 cell viability, proliferation, migration/invasion, and promotes apoptosis. DEX upregulates miR-381, which targets LRRC4, thereby activating the SDF-1/CXCR4 signaling pathway	Apoptosis	Systematically studies multi-faceted protective effects of DEX (viability, proliferation, migration)	Lack of *in vivo* validation	(73)
PD	MPP?, SH-SY5Y cells	Attenuates loss of mitochondrial membrane potential, reduces ROS, decreases Cleaved Caspase-3/9 activation	Apoptosis	Explores novel mechanisms in a PD model	Lack of *in vivo* validation	(59)
PD	MPTP, mice	Activates AMPK, enhances PINK1/Parkin-mediated mitophagy, improves mitochondrial function, reduces oxidative stress, upregulates Bcl-2, downregulates Bax and Cleaved Caspase-3/9	Apoptosis, autophagy (mitophagy)	Improve disease function	Does not distinguish contributions of each pathway in different cell types	(75)
PD	MPTP, mice	Upregulates Bcl-2, downregulates Bax, Cleaved Caspase-3/9 in the substantia nigra. Reduces neuroinflammation (NF-κB pathway)	Apoptosis	Demonstrates regulation of classic apoptotic proteins, linked to anti-inflammatory effect	Relatively traditional mechanistic exploration	(74)
AD	Aβ, SH-SY5Y cells	Promoted HSPB8 expression via inhibiting the lncRNA SNHG14/UPF1 axis to inhibit nerve cell apoptosis	Apoptosis	Reveals a novel regulatory mechanism involving non-coding RNA	Not validated in complex AD animal models	(69)
AD	Aβ, mice	Reduces LC3-II/p62 accumulation, promotes autophagosome-lysosome fusion, and alleviates memory impairment	Autophagy	Fluorescence localization	Not validated in complex AD animal models	(87)
Neonatal hypoxic-ischemic brain injury	Modified HIBI model, 7-day-old rats	Inhibits autophagy in neurons and microglia, downregulates LC3-II, Beclin1, reduces neuronal loss and demyelination, and improves long-term neurological outcomes. Effect is reversed by rapamycin	Autophagy	Confirms the inhibitory effect on excessive autophagy and its functional relevance	Upstream signals for autophagy inhibition are not elucidated	(79)
Developmental brain injury	Sevoflurane, rats	Antagonizes sevoflurane-induced blockade of autophagic flux (downregulates LC3, p62) and abnormal mitochondrial dynamics (downregulates Drp1, Bax, upregulates Bcl2)	Autophagy	Focuses on the special vulnerability of the developing brain	High model specificity	(80)
Cognitive dysfunction	Sevoflurane, aged rats	downregulates LC3-II, Beclin-1 and ameliorates cognitive dysfunction	Autophagy	An aged animal model	Primarily correlative	(81)
Anesthetic neurotoxicity	Propofol, primary hippocampal neurons	Downregulates HOXA5 expression, inhibits NLRP3 inflammasome activation, reduces levels of GSDMD-N, cleaved-caspase-1, IL-1β, and IL-18.	Pyroptosis	A novel mechanism for HOXA5-NLRP3	Lacks functional validation in animal models	(92)
Anesthetic neurotoxicity	Ropivacaine, SK-N-SH cells	Promotes Nrf2 nuclear translocation and enhances HO-1 expression. Inhibits NLRP3 inflammasome activation and downstream pyroptosis markers	Pyroptosis	Clearly connects the antioxidant pathway (Nrf2/HO-1) with pyroptosis inhibition	Lack of *in vivo* validation	(94)
Anesthetic neurotoxicity	Bupivacaine, SH-SY5Y cells	Upregulates miR-7-5p, which targets and inhibits PARP1 expression, reversing MMP collapse and ROS accumulation	parthanatos	Provides a direct molecular mechanism	Lack of *in vivo* validation	(124)
Perioperative neurocognitive disorders	PND model, aged mice	Improves cognitive impairment and anxious behavior. Reduces hippocampal inflammation and neuron pyroptosis (GSDMD, NLRP3, IL-1β, IL-18). Upregulates miR-184-3p.	Pyroptosis	Identifies a novel regulatory node	Lack of downstream mechanism exploration	(95)
ICH	ICH model, mice	Regulates iron metabolism, amino acid metabolism and lipid peroxidation processes to inhibit ferroptosis	Ferroptosis	Covers three levels: iron metabolism, amino acid metabolism, and lipid peroxidation	There was no clear association with known core ferroptosis pathways (e.g., GPX4, System Xc-)	(104)
Spinal cord injury	Lidocaine, rats, PC12 cells	Upregulates CISD2, FTH1, SLC7A11, GPX4; downregulates NCOA4. CISD2 inhibition reverses DEX's protective effects	Ferroptosis	Clarifies a novel mechanism: DEX inhibits ferroptosis by upregulating CISD2 to suppress ferritinophagy	Limited exploration of the downstream execution of ferroptosis	(105)
Spinal cord injury	Lidocaine, rat; SK-N-SH cells	Inhibits PKC-δ phosphorylation, pyroptosis (reduced caspase-1, NLRP3, ASC), and pro-inflammatory cytokine (IL-1β, IL-18) expression. Attenuates microglial response	Pyroptosis	Proposes a novel mechanism	Lacks specific causal verification of the PKC-δ pathway	(93)
AD	Aβ, mice; HT22 cells	Activates mTOR-TFR1 signaling, upregulates SLC7A11 and GPX4. Inhibition of mTOR blocks this process and improves cognitive function in AD mice	Ferroptosis	Reveals the relatively novel mTOR-TFR1 axis in ferroptosis regulation	Does not elucidate how mTOR precisely regulates TFR1 and downstream proteins	(106)
sepsis	CLP, mice	Activates spinal astrocyte α2A-AR, inhibits production of inflammatory factors (C3, IL-6, TNF-α), and reduces necroptosis of spinal GABAergic neurons	Necroptosis	Reveals a novel neuroimmune pathway	Only focusing on the spinal cord mechanism	(116)

DEX, dexmedetomidine; PCD, programmed cell death; I/R, ischemia/reperfusion; OGD/R, oxygen-glucose deprivation/reoxygenation; MCAO, middle cerebral artery occlusion; MMP, mitochondrial membrane potential; ERS, endoplasmic reticulum stress; TBI, traumatic brain injury; ICH, intracerebral hemorrhage; CPB, cardiopulmonary bypass; LPS, lipopolysaccharide; PD, Parkinson's disease; AD, Alzheimer's disease; PND, perioperative neurocognitive disorders; SCI, spinal cord injury.

### 3.1 Apoptosis

Apoptosis is a process of orderly cell death that is characterized by cell contraction, condensation of chromatin, DNA cleavage in the interkaryon, reorganization of the cytoskeleton, and the formation of apoptotic bodies (53). Apoptosis plays a crucial role in the development, tissue homeostasis and diseases of organisms, and its regulatory mechanisms involve two types: the endogenous pathway (mitochondrial pathway) and the exogenous pathway (death receptor pathway) (9). Numerous studies have reported that DEX can reduce neuronal apoptosis in *in vivo* and *in vitro* models of IS and ICH. The underlying mechanisms include: regulating mitochondrial function, inhibiting oxidative stress, inhibiting endoplasmic reticulum stress (ERS), and mediating key signaling pathways.

The mitochondrial membrane potential (MMP) is an electrical potential difference caused by ion concentration disparities across the mitochondrial inner membrane. MMP stability is essential for mitochondrial function. Pathologically, the MMP decreases, releasing cytochrome c and activating the cysteine protease cascade, ultimately leading to apoptosis (54). Wu et al. (55) developed an *in vitro* model of cerebral I/R and demonstrated that DEX treatment mitigated the reduction in the matrix MMP in neuro-2a cells. This mechanism inhibits the release of cytochrome c from mitochondria into the cytoplasm, reduces caspase-9/3/6 activation, and consequently decreases apoptosis. Another study similarly revealed that DEX enhances MMP expression by regulating mitochondrial membrane permeability through calcium overload inhibition in neurons, thereby reducing glutamate-induced neuronal apoptosis (56).

In the PD model, DEX has shown potent protective effects, as shown in both cellular and animal PD models (57, 58). Notably, DEX has a role in mediating apoptosis in PD. For example, Chen et al. confirmed that DEX can significantly alleviate the decrease in mitochondrial membrane potential in MPP-induced SH-SY5Y dopaminergic neuronal cell models, thereby inhibiting cell apoptosis (59).

Nrf2, a key intracellular transcription factor, plays a crucial role in apoptosis by regulating oxidative stress and antioxidant responses. When cells are exposed to oxidative stress, Nrf2 is translocated into the nucleus, binds to the antioxidant response element (ARE), and activates the expression of downstream antioxidant genes such as heme oxygenase-1 (HO-1) and NAD(P)H: quinone oxidoreductase 1 (NQO-1) (60). Xu et al. revealed that DEX enhances the expression of downstream antioxidant genes, including HO-1, NQO1, and SOD in an OGD/R-induced cell model. DEX treatment upregulated the Nrf2/ARE pathway by activating α2-AR. Co-immunoprecipitation confirmed the direct binding between α2-AR and Nrf2. Downregulating Nrf2 by Nrf2-targeted siRNA significantly reversed the protective effect of DEX (61). Similar mechanisms have been reported in animal models of TBI (36) and cerebral hemorrhage (62). DEX activates the Nrf2 pathway to initiate antioxidant mechanisms and inhibit neuronal apoptosis. In the glutamate-induced neuronal injury model, DEX can reduce the levels of oxidative mediators (e.g., ROS and MDA) and inhibit neuronal apoptosis (56).

When exposed to pathological conditions like ischemia, hypoxia, oxidative stress, misfolded or unfolded proteins accumulate, triggering ERS, which activates downstream apoptosis pathways, ultimately leading to cell death (63). ERS is a critical component in the pathophysiology of TBI, contributing to secondary damage through multiple mechanisms (64, 65). Studies have shown that DEX significantly reduces neuronal apoptosis in TBI mice by inhibiting the expression of ERSS-related apoptosis proteins (e.g., CHOP and caspase-3), thereby improving neurological function scores and reducing secondary brain damage (37). In cerebral I/R injury models, DEX inhibits the PERK–CHOP–caspase-11 signaling pathway, thereby reducing the expression of ERS markers (e.g., GRP78, p-PERK, and CHOP) and ultimately decreasing ERS-mediated neuronal apoptosis (66). DEX can also inhibit the expression of ERS-associated apoptosis proteins (e.g., caspase-3 and CHOP) by activating the Sigma-1 receptor, thereby reducing neuronal apoptosis during cerebral I/R injury (67).

DEX achieves an anti-apoptotic ability through affecting several signaling pathways. Chen et al. (68) confirmed that DEX reduces neuronal apoptosis in the hippocampus and cerebral cortex of rats induced by CPB surgery, with its antiapoptotic mechanism likely involving inhibition of the JAK2-STAT3 pathway. *In vitro* studies have shown that DEX can activate the HIF-1α/p53 signaling pathway, thereby protecting hippocampal neurons from hypoxia/reoxygenation-induced apoptosis (20). In the AD model, DEX inhibits the effect of UPF1 on HSPB8 mRNA stability by downregulating the expression of the long noncoding RNA SNHG14, thereby reducing neuronal apoptosis (69). Studies have shown that in LPS-induced neural injury models, DEX suppresses the p38 MAPK pathway (rather than the JNK and ERK pathways), decreases the expression of apoptosis-related proteins such as BAX, and inhibits hippocampal neuronal apoptosis (70). Additionally, DEX can modulate miRNAs to exert antiapoptotic effects on neurons. For example, DEX reduces propofol-induced hippocampal neuronal damage and HT22 apoptosis by regulating the miR-377-5p/Arc pathway (71). In IS models (72) and ropivacaine-induced neural injury models (73), DEX inhibits neuronal apoptosis by regulating miR-381 expression. In PD models, the regulation of classical apoptosis-executing proteins by DEX represents one of its key protective mechanisms. Zhang et al. demonstrated in an MPTP-induced PD mouse model that DEX treatment markedly upregulated the anti-apoptotic protein Bcl-2 and downregulated the pro-apoptotic proteins Bax, Cleaved Caspase-3, and Cleaved Caspase-9, thereby reducing the loss of dopaminergic neurons in the substantia nigra pars compacta and improving motor function (74). Chen et al. revealed that in MPTP-induced PD mice, DEX enhanced mitophagy via AMPK activation. Therefore, DEX downregulated apoptosis-related proteins (Bax and cleaved Caspase-3/9), exerting an anti-apoptotic effect (75).

### 3.2 Autophagy

Autophagy refers to a cellular degradation process that transports necrotic organelles and other cellular debris to lysosomes via autophagosomes, playing a vital role in maintaining intracellular homeostasis (76). The process comprises four stages: initiation, phagosome elongation and autophagosome formation, autophagolysosomal fusion, and substrate degradation, which are regulated by mTOR, autophagy marker proteins (e.g., Beclin1, LC3, p62), and other autophagy-related proteins (77). However, excessive autophagy can induce cell death and exacerbate cellular damage (78). In a hypoxic-ischemic brain injury (HIBI) model, LC3 immunofluorescence staining showed that DEX similarly inhibited the overactivation of autophagy in neurons and microglia, improving the neurological function of rats (79). In models of neurotoxicity induced by sevoflurane in aged and neonatal rats, DEX similarly alleviates cognitive impairment and neural damage in the animal models by modulating autophagy-related proteins (such as LC3-II and Beclin-1) (80, 81). Studies have shown that in cerebral I/R injury models, DEX has neuroprotective effects through upregulating HIF-1 expression, suppressing beclin-1 expression, and reducing neuronal autophagy (82, 83). Zhu et al. (84) reported that DEX has neuroprotective effects on middle cerebral artery occlusion (MCAO) rats via autophagy regulation, but these effects are reversed by JNK pathway activators, indicating that DEX can inhibit JNK pathway-mediated neuronal autophagy. In cerebral I/R injury models, DEX reduces neuronal damage by suppressing miR-199a expression and decreasing autophagy-related protein expression (85). In TBI models, DEX reduces the protein expression of LC3, beclin-1, NF-κB, and proinflammatory factors by regulating the ROS/Nrf2 signaling pathway, thereby increasing neuronal survival rates (19).

Dysregulated neuronal autophagy and abnormal protein aggregation/folding are the primary pathological features of NDDs (86). Lee et al. reported that in AD animal models, treatment with DEX can effectively ameliorate dysfunction in the autophagy-lysosome pathway. It reduces the abnormal accumulation of the autophagosome marker protein LC3-II and the substrate p62, and promotes the fusion of autophagosomes with lysosomes, converting double-membrane autophagosomes into single-membrane autolysosomes, thereby restoring autophagic flux and improving memory deficits (87). DEX shows protective effects in PD by modulating autophagy. In the MPTP-induced PD mouse model, DEX was shown to activate the AMPK signaling pathway, thereby potentiating PINK1/Parkin-mediated mitophagy. DEX administration increased the number of mitophagosomes, enhanced the LC3-II/I ratio and p62 degradation, restored mitochondrial complex activity, and dampened oxidative stress, thus mediating a protective effect of dopaminergic neurons in the substantia Nigra (75).

Additionally, DEX can promote the clearance of harmful substances by enhancing or restoring autophagic flux. In *in vitro* OGD/R models, DEX enhances autophagy activity and improves cellular viability and mitochondrial function by increasing SIRT3 expression (88).

These studies indicate that DEX can precisely bidirectionally regulate neuronal autophagy either by directly modulating autophagy-related proteins or through various signaling pathways such as oxidative stress, microRNAs, HIF-1α/Beclin1, and AMPK/PINK1/Parkin. However, the regulatory roles of DEX in autophagy might depend on specific pathological contexts. In AD and PD models, DEX promotes the clearance of toxic proteins and damaged organelles by enhancing or restoring protective autophagy (including mitophagy). In acute CNS disorders, such as IS and TBI, DEX inhibits excessive or dysfunctional autophagy, thus preventing secondary neuronal death.

### 3.3 Pyroptosis

Pyroptosis is an inflammatory PCD process characterized by distinct morphological (including chromatin condensation and DNA fragmentation) and pathophysiological alterations (89). Normally, pyroptosis is activated under the stimulation of exogenous pathogen-associated molecular patterns (PAMPs) and endogenous damage-associated molecular patterns (DAMPs). Then, active Caspase 1 is cleaved from pro-Caspase1 through apoptosis-associated speck-like protein containing a CARD (ASC). Active Caspase-1 can not only cleave the precursor proteins of proinflammatory cytokines (such as IL-1β and IL-18) into their active forms, but also cleave proteins of the gasdermin family (e.g., GSDMD) to form active *N*-terminal domains. Finally, pyroptosis contributes to activation of inflammatory caspases, cell membrane rupture, cell swelling, and the production of proinflammatory cytokines and other cytoplasmic contents (89, 90). Pyroptosis exerts a crucial regulatory role in the initiation and development of neurological diseases (91).

The roles of DEX in mediating pyroptosis in CNS disorders been increasingly noted. For example, DEX exhibits neuroprotective effects through inhibiting NLRP3 inflammasome activation. DEX downregulates HOXA5 expression and inhibits HOXA5 binding to the NLRP3 promoter region. These mechanisms reduce NLRP3 expression, lower the protein levels of GSDMD-N and cleaved caspase-1, and decrease the release of the inflammatory cytokines IL-1β and IL-18, ultimately significantly alleviating neuronal pyroptosis (92, 93). Studies have shown that DEX activates the Nrf2/HO-1 pathway to suppress NLRP3 expression, thereby mitigating ropivacaine-induced neuronal pyroptosis (94). Additionally, DEX upregulates miR-184-3p expression to inhibit NLRP3 inflammasome activation, further reducing pyroptosis and neuroinflammation (95). Furthermore, DEX enhances mitochondrial autophagy by activating the PINK1 pathway, which inhibits caspase-1/11-GSDMD-dependent neuronal pyroptosis (96, 97).

### 3.4 Ferroptosis

Ferroptosis represents a newly identified regulated form of cell death whose core characteristics include iron ion accumulation, lipid peroxidation, and cell membrane damage (98). The essence of ferroptosis lies in the depletion of GSH and the subsequent decrease in the activity of GPX4. Lipid peroxides cannot be metabolized through the glutathione reductase reaction catalyzed by GPX4; subsequently, divalent iron ions oxidize lipids to ROS, which leads to cell membrane destruction and thereby induces ferroptosis (99). In recent years, ferroptosis has been found to be closely associated with the pathological processes of brain damage and NDDs (100, 101).

Hu et al. (102) confirmed that DEX treatment improved neurobehavioral function and decreased the cerebral infarction volume in MCAO mice. Further mechanistic studies revealed that DEX alleviates mitochondrial shrinkage in MCAO mouse brains, decreases MDA and ferrous ion (Fe2+) levels, and elevates the GSH content. It also upregulates the expression of Nrf2 and ferroptosis-related proteins (GPX4 and SLC7A11) in stroke models. Bioinformatics analysis revealed that four HIF-1 signaling pathway genes (HMOX1, STAT3, CYBB, and TLR4) associated with ferroptosis are closely linked to ischemic stroke pathogenesis (103). In MCAO mice, the expression of these four genes was significantly reduced. DEX treatment reversed these changes, significantly reduced cell death, improved neurobehavioral deficits, and lowered the levels of serum and brain inflammatory factors (TNF-α and IL-6) and oxidative stress mediators (MDA and GSSG) (103). In cerebral hemorrhage models, DEX was found to reduce iron ion and MDA levels while increasing GPX4 and GSH levels, thereby inhibiting neuronal ferroptosis (104). Studies have revealed that DEX upregulates the expression of CDGSH iron–sulfur domain 2 (CISD2) in a rat model of spinal cord injury and enhances FTH1, SLC7A11, and GPX4 expression to suppress ferritin autophagy-mediated ferroptosis (105). Li et al. (106) reported that DEX activates the mTOR-TFR1 pathway, significantly elevates SLC7A11 and GPX4 protein levels, and reduces hippocampal neuronal ferroptosis in AD mice. Another study demonstrated that DEX can downregulate the expression of SOX9, thereby inhibiting the transcriptional activity of DMT1. This mechanism reduces iron absorption and accumulation, ultimately decreasing iron-induced apoptosis in neurons in the brain I/R model (107). On the basis of the above studies, DEX clearly plays a neuroprotective role in brain injury and AD by suppressing neuronal ferroptosis, which is achieved through direct regulation of iron metabolism, mitigation of oxidative stress, and attenuation of lipid peroxidation.

Neuronal ferroptosis arises not only from intrinsic metabolic dysregulation but is also profoundly influenced by glial-neuronal interactions. Emerging evidence highlights that glial crosstalk serves as a key inducer of neuronal ferroptosis in PD. Oligodendrocytes and astrocytes can abnormally activate fibroblast growth factor (FGF) signaling, upregulating ferroptosis-related genes and exacerbating damage to substantia nigra dopaminergic neurons (108). This study expands the regulatory perspective of ferroptosis from the neuron itself to the neural microenvironment, underscoring the potential of targeting glial function or intercellular communication as a novel neuroprotective strategy.

DEX has shown potent functions in modulating neuroinflammation and glial activation, which have a great impact on neuronal ferroptosis (109). For instance, in cerebral ischemia-reperfusion injury models, DEX has been shown to promote a shift of microglia toward the protective M2 phenotype via specific signaling pathways, thereby attenuating neuroinflammation (82). In a PD-related pain model, DEX effectively suppressed abnormal activation of spinal astrocytes and the release of pro-inflammatory factors (110). Although these studies did not directly examine measures of ferroptosis, chronic neuroinflammation and activated glial cells, particularly the proinflammatory phenotype, are key drivers of oxidative stress, disordered iron metabolism, and lipid peroxidation. Therefore, anti-inflammatory therapy targeting glial cells is expected to indirectly combat neuronal ferroptosis by remodeling the microenvironment, which reveals a potential new target and action dimension for the neuroprotective effect of DEX in PD and related CNS.

### 3.5 Necroptosis

Necroptosis serves as an alternative cell death pathway that is triggered when apoptosis is impaired, with its characteristics including organelle swelling, plasma membrane rupture, and the release of cellular contents. Which induces an inflammatory response (111). Its occurrence is determined by factors such as the severity of injury, ischemia, edema, and secondary inflammatory responses and is comediated by RIPK1, RIPK3, and MLKL (9). Necroptosis promotes neuronal death and neuroinflammation, which play crucial roles in the pathological processes of NDDs and brain injury (112, 113). Studies have reported that DEX can effectively inhibit cardiomyocyte necroptosis in myocardial I/R injury (114, 115). A study by He et al. (116) revealed that in a sepsis-induced cardiomyopathy model, excessive activation of peripheral sympathetic nerve excitability and necroptosis of spinal GABAergic neurons were observed, manifested by the upregulation of necroptotic effector genes such as RIPK1, RIPK3, and MLKL. DEX treatment significantly activated the α2A-adrenergic receptor in spinal astrocytes and markedly inhibited the production of inflammatory factors (e.g., C3, IL-6, and TNF-α), thereby reducing necroptosis of GABAergic neurons and alleviating sepsis-related cardiac dysfunction (116). This study revealed that the inhibitory effect of DEX on GABAergic neuronal necroptosis is mediated by the activation of the α2A-adrenergic receptor in spinal astrocytes.

### 3.6 Parthanatos

Parthanatos is a novel form of PCD triggered by excessive activation of poly (ADP–ribose) polymerase-1 (PARP-1), distinct from caspase-dependent apoptosis (117). Under physiological conditions, the basal activity of PARP-1 is crucial for maintaining genomic stability and mediating DNA repair, serving as a guardian of cell survival (118). When PARP-1 is overactivated, it consumes large amounts of nicotinamide adenine dinucleotide (NAD) and adenosine triphosphate (ATP), catalyzing the formation of a large amount of PAR polymers (119). These aggregates can induce AIF to translocate from the mitochondria to the nucleus, and the AIF released into the cytoplasm binds with MIF to form the AIF/MIF complex, which is then transported to the nucleus, causing extensive DNA fragmentation and ultimately leading to cell death (119).

Parthanatos is a key common pathway connecting DNA damage, oxidative stress, mitochondrial dysfunction, and neuroinflammation, and it is widely involved in the pathological processes of various CNS diseases, including AIS (120) and PD (121). For instance, PARP-1 inhibitors have been shown to block the toxicity of pathological α-synuclein in PD mouse models (122). Given that direct PARP-1 inhibition may interfere with its physiological DNA repair functions, current research focuses on developing downstream-specific targets, such as inhibitors of MIF nuclease activity, a strategy that shows potential in preventing neurodegeneration in PD models (123).

With increasing research on how DEX regulates neuronal PCD, studies have clarified the effect of DEX on neuronal parthanatos. Zheng et al. (124) demonstrated that in a bupivacaine-induced SH-SY5Y neuroblastoma cell injury model, DEX upregulates the expression of miR-7-5p, which targets and inhibits the3′ UTR of PARP1. This reduces excessive activation of PARP1, effectively reverses bupivacaine-induced mitochondrial membrane potential collapse and ROS accumulation, and ultimately inhibits the occurrence of neuronal Parthanatos. This study revealed that DEX can inhibit oxidative stress and mitochondrial dysfunction, and prevent neuronal parthanatos by regulating the miR-7-5p/PARP1 axis.

Parthanatos-associated targets provide potential strategies for neuroprotective interventions. Future therapeutic strategies may shift from traditional PARP-1 inhibition to the development of specific inhibitors for downstream effector molecules (such as MIF), or to finding indirect protectants that can stabilize mitochondria and regulate related signaling pathways. DEX has antioxidant properties and can protect mitochondrial function, which is closely related to the regulation of upstream events in parthanatos. It can also regulate cellular parthanatos through the miR-7-5p/PARP1 axis. Therefore, further investigation into whether DEX can inhibit parthanatos in disease models such as IS or PD, and elucidating its specific mechanisms of action, will be an important direction for refining its multi-target neuroprotective network.

### 3.7 Coordinated regulatory network

The previous sections described the regulatory effects of DEX on various PCD pathways, including apoptosis, autophagy, and ferroptosis, respectively. There are significant intersections between two of those PCD pathways (Table 1). Such an interconnected network is often driven by common upstream stress factors (e.g., oxidative stress, mitochondrial damage, inflammation). Their interactions are not isolated, forming a complex regulatory network characterized by “upstream signal sharing, midstream molecular crossover, downstream effect antagonism or synergy.” Under normal physiological conditions, the antagonistic effect between PCD pathways is dominant, thus avoiding excessive cell death. For example, protective autophagy can inhibit other PCD pathways and maintain cell survival. Under pathological conditions (e.g., infection and ischemia-reperfusion), the balance is disrupted, and the coordinated/abnormal switching of the PCD pathway can lead to tissue damage and inflammation amplification. Autophagy transforms into lethality, forming a death amplification effect. Additionally, the activation of different PCD pathways is closely related to the activation of key molecules. For example, the activation/inactivation of core molecules such as caspase-8, caspase-3, GPX4, and PARP1 directly determines the switching of different PCD pathways and is a core target for regulating cell death fate.

Studies have shown that DEX can activate core antioxidant pathways such as Nrf2/ARE and AMPK, enhance the activity of endogenous defense systems such as SOD and GSH, and effectively reduce ROS and MDA levels (19, 56, 75). At the same time, it reduces the release of pro-inflammatory factors (such as IL-1β) by inhibiting the NLRP3 inflammasome and the classical inflammatory signaling pathway, such as NF-κB (88, 89). Together, these effects stabilize mitochondrial function and reduce factors contributing to DNA damage and energy crises. DEX can improve the intracellular environment by eliminating oxidative stress and inflammation, and weaken the upstream initiation signals of apoptosis, pyroptosis, ferroptosis, and abnormal autophagy. Thus, the complex pathology in different disease models, such as NDDs, involves the superposition of multiple PCDs (Table 3). DEX has been confirmed to affect both apoptosis and autophagy-related pathways in the PD model. This suggests that DEX does not target a single form of death, but rather remodels the overall neuronal death decision network in response to proteotoxicity, oxidative damage, and inflammatory stress through the above-mentioned multi-target strategy, thereby maintaining cell survival more effectively.

In addition, DEX also regulates PCD multiplex by targeting key signaling nodes. For example, in PD, DEX-activated AMPK signaling can both enhance protective mitophagy (removal of damaged mitochondria) through PINK1/Parkin (75) and inhibit the mitochondrial pathway of apoptosis by regulating Bcl-2/Bax balance (75). Nrf2 is an important transcription factor during oxidative stress. On the one hand, DEX inhibits oxidative stress and reduces the expression of autophagy-related proteins by activating Nrf2 expression. On the one hand, it inhibits pyroptosis by inhibiting the activation of NLRP3 inflammasome (19, 90). This mode of simultaneous regulation of multiple downstream pathways through a key signaling node is the core embodiment of DEX's synergistic protection.

Taken together, the neuroprotective mechanism of DEX cannot be attributed to a simple inhibition or activation of a single PCD pathway. It regulates neuronal programmed death through a highly interconnected signal network with multiple targets and coordination. The core of its mechanism is to play anti-inflammatory and anti-oxidative stress effects through AMPK and Nrf2 pathways, regulate autophagic flow, and cooperate with anti-apoptosis and anti-pyroptosis effects to finally maintain neuronal mitochondrial function and overall cell homeostasis.

## 4 Conclusion and perspectives

**Figure 2:**
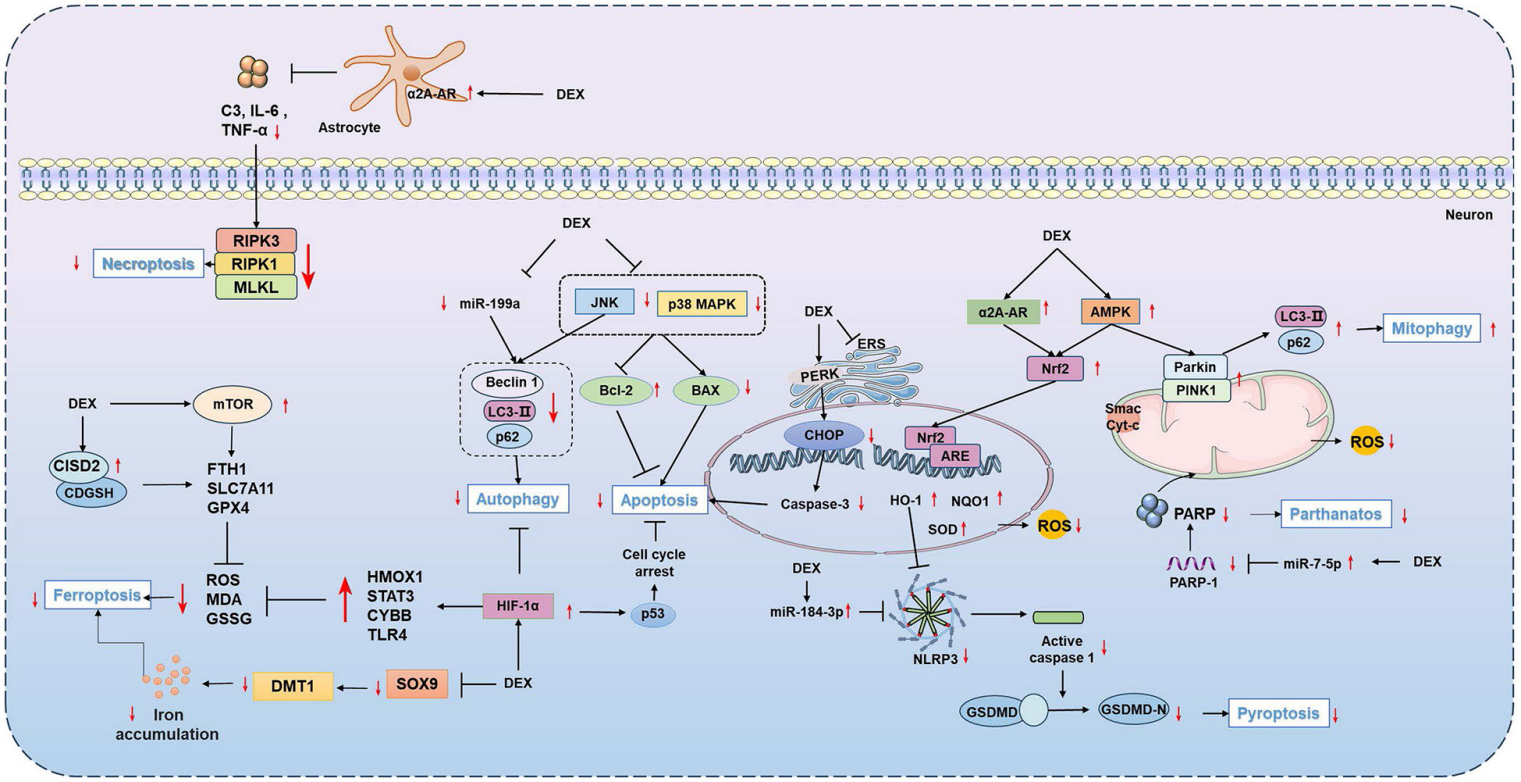
Molecular mechanisms of Dex-mediated neuronal protection via mediating PCD pathways. This schematic illustrates the principal molecular targets and signaling pathways by which DEX confers neuroprotection across various CNS disorders. DEX, Dexmedetomidine; PCD, Programmed Cell Death; ERS, Endoplasmic Reticulum Stress.

In conclusion, DEX has significant neuroprotective effects on various CNS disorders. Specifically, DEX mitigates neuronal damage induced by pathological stimuli by modulating multiple PCD pathways, including apoptosis, autophagy, pyroptosis, ferroptosis, necroptosis, and parthanatos. These regulatory mechanisms may involve enhancing mitochondrial function, suppressing oxidative stress, and regulating miRNA expression (Figure 2).

PCD is a gene-regulated self-destruction process initiated by cells, which includes apoptosis, autophagy, pyroptosis, ferroptosis, and necroptosis. Neuronal PCD has been proven to play a central role in the pathological mechanisms of NDDs and brain injury-related diseases (9, 125). As research on PCD in various pathological processes has advanced, targeted PCD therapies have emerged as promising treatment strategies (9, 126, 127). For example, a nanodrug delivery system based on the biomimetic delivery of macrophage membrane carrying minocycline can specifically inhibit neuronal pyroptosis by down-regulating GSDMD-N, caspase-1, IL-1β, and IL-18 in SCI (128). This review first summarizes the clinical protective evidence of DEX in CNS diseases, and then systematically elaborates the core molecular mechanism of DEX's protective effect by regulating PCD pathways in a variety of neurons. A central insight revealed in this review is that the neuroprotective effects of DEX are not dependent on the regulation of a single isolated pathway. Multiple PCD pathways form a highly interconnected regulatory network through shared upstream events (e.g., oxidative stress, mitochondrial damage) and key hub molecules (e.g., AMPK, Nrf2). DEX is playing a synergistic regulatory role by acting on these core nodes simultaneously. For example, its activation of AMPK both enhances protective mitophagy and inhibits mitochondrial pathway apoptosis in a PD model. This integrative perspective suggests that future research on the mechanism of DEX should go beyond a single pathway and focus on how DEX dynamically rebalances the whole PCD network in different disease backgrounds, including the relative contribution of each pathway and activation timing.

Despite the wealth of preclinical evidence, limitations remain. Studies have shown that DEX is neurotoxic at high doses, such as rats injected with more than 5 μg/kg of DEX increase neuronal apoptosis (129). Future neuroprotective research should focus on establishing maximum safe dosages of DEX and exploring combination therapies with other neuroprotective agents. PCD pathways (e.g., cuproptosis and disulfideptosis) also play pivotal roles in NDDs, and their mechanisms have been elucidated (130). Further investigations are warranted to confirm whether the neuroprotective effects of DEX are mediated by these novel PCD pathways. Moreover, neuronal death typically involves complex crosstalk among multiple PCD pathways. For example, in IS, inhibiting apoptosis may increase the risk of necroptosis, whereas balanced regulation enhances neuronal survival rates (131). Future investigations into how DEX modulates critical cross-talk networks among different PCD pathways will offer a more in-depth understanding of its mechanisms in regulating neuronal PCD across various diseases.

At the level of clinical translation, the challenges are more pronounced. For clinical studies, most studies have used DEX as the preferred sedative for patients with brain injury during the perioperative period or in the ICU, and there are few reports on its direct therapeutic effect. Some studies have begun to focus on the preventive effect of DEX on specific neurological complications, such as PSH (43). Most of the existing clinical studies are retrospective and single-center designs, which might have time sequence bias, statistical underpower, and a lack of long-term prognosis data. In the future, well-designed, high-quality prospective clinical trials with patient-centered outcomes as the primary endpoint are needed to confirm the long-term protective value of DEX on neurological function in patients.
